# Modern Medico-Psychology and Psychiatry

**Published:** 1894-01-27

**Authors:** T. S. Clouston

**Affiliations:** Lecturer on Mental Diseases, Edinburgh University, and Physician Superintendent Royal Asylum, Morningside


### MODERN MEDICO-PSYCHOLOGY AND PSYCHIATRY.

By T. S. CLOUSTON, M.D., Lecturer on Mental Diseases, Edinburgh University, and Physician Superintendent Royal Asylum, Morningside.

### I.—INTRODUCTORY.

THE history of disease from the evolutional point of view has been one long effort to substitute for the animal and savage practice of slaughter and special neglect of the sick and hurt, the duty of special care, study, and help. Nature has unquestionably implanted feelings of aversion and even loathing in the sound towards the subjects of many diseases. The steady untiring aim of all the really good men and women of all the religions worth the name, of many legislators, of all the followers of the healing art, of all the hospital and asylum builders, and of the nursing fraternities in all ages, has been to eradicate such abhorrence, to rouse pity, to excite interest, and to give help with a view to cure those who were the subjects of disease. Almost no strong and healthy young man of twenty but has a natural shrinking from disease and would neglect it. Such feelings exist, and must be reckoned with; they are part of the instinctive feelings of mankind. Towards no class of diseases have those feelings of aversion and abhorence been so strong as in the case of mental disease. "Madness" has afforded to literature a whole terminology of loathing. Superstition, exaggeration, and fear seemed to run riot whenever the subject came up. The only grain of comfort for the mentally afflicted of old was provided by the superstitious belief of certain Eastern peoples, that they were under the special protection of the Deity, and so must be indulgently treated. Unfortunately early Christianity took the opposite view that they were possessed by devils, and so ought to be harshly dealt with. Scarcely more than a hundred years have elapsed since first medical science and philanthropy combined in the persons of Pinel in France, and William Tuke, the Quaker of York, to take an entirely different view of mental disorders. Those men propagated their ideas among mankind, and embodied them practically by a more careful study and an improved treatment of the insane in the Bicêtre Hospital in Paris, and in the "Retreat" at York. It did not take long for knowledge on the subject to diffuse itself among the thoughtful and the good, and for the general conscience to be awakened. The next step was naturally practical effort by legislation to provide hospitals for the insane—unfortunately as it has turned out—called "Lunatic Asylums." What the late Lord Shaftesbury did for the mentally diseased by passing the Lunacy Acts for England in 1845, whereby in every county an hospital has been established, and by instituting the great inspecting and protecting machinery of the permanent Lunacy Commission, it is impossible to narrate in any short series of articles. In fifty years a disease very prevalent, very neglected, very pitiable has been provided for at an enormous cost by the public. Certainly over £15,000,000 has been spent on means of care and treatment scarcely accorded to any other disease on so large and systematic a scale. From being the outcast of society in the eighteenth century, the "madman" has become the child of the State in the nineteenth—housed, fed, and doctored at the public expense, and all who have to do with him inspected and held rigorously accountable to a special department of the State. Now at the end of the century the disease is regarded as of so high importance that skilled special pathologists are paid for out of the public funds, and provided with laboratories in which to investigate the altered condition of the brain structure, and to throw light on its causes by every means known to modern science. So great a revolution in feeling and in practice has scarcely ever taken place in regard to any medical and social question within so short a time. It was soon discovered that the disease had a high value, and an interest from the scientific point of view; and out of its study has arisen modern medico-psychology, "psychiatry" or "alienism;" unfortunately we have no equivalents in English for those German and French terms. In these articles I shall use "medico-psychology" to cover the study, treatment, and pathology of mental diseases and not in its more restricted sense physiological psychology, and experimental psychology.

Looked at from the human and social point of view, the undoubted fact that no other disease approaches mental disorder in the terror it inspires, the sense of hopelessness it causes in the uninstructed, the deep distress to relatives of the patients and the upsetting of social ties, largely accounts for its not being studied or its true nature understood, and for its treatment being barbarous till recent times. But the modern scientific spirit did not allow this field to lie fallow, and its study was hardly begun, when its profound interest and importance were seen by certain physicians. The great problems thus opened up of the relationship of mind to body, have exercised a fascination over many of the greatest minds in our profession during the past hundred years, men in many cases whose professional work was not specially to treat mental diseases. I need only mention the names of Pinel, Esquirol, Bayle, Falret, Brierre de Boismont, Ball and Charcot in France; Calmeil in Belgium; Van der Kolk in Holland; Jacobi, Griesinger, Feuchtersleben, Meynert, Westphal, and Krafft-Ebing in Germany; Tamburini and Lombroso in Italy; Rush, Ray, Bell, Hammond, Spitzka in America; and Abercrombie, Prichard, Combe, Conolly, Brodie, Holland, Laycock, Maudsley, Bucknill, Tuke, and Bevan Lewis in this country. In each of those countries there are now scientific journals devoted to this department, with hundreds of able observers contributing new articles and making new observations every year.

It was soon discovered by those men that mental disease and mental physiology had to be studied in the same inductive way as all diseases and all physiology must be studied. Observation and experiment aided cautiously by subjective experience, and by hypothesis, with sound induction based on the facts and on them alone, were the methods employed. Ceaseless clinical work, careful experiments in modes of treatment and their effects, and exact researches into the state of the brain after death have, up to this time, produced more results by far than all the previous speculations as to the nature of insanity, from the purely mental and introspective point of view. It has always been the earnest hope of those who have interested themselves in mental diseases, either from the scientific or the philanthropic point of view, that a more thorough knowledge of their nature and treatment would in time lead to a diminution of their total amount. The most casual study of them shows them to be very hereditary in their character, and naturally it is supposed that if the persons who by inheritance have a tendency to them, can be subjected during the period of development and consolidation of function of the brain, to the right sort of hygienic and preventive measures, the amount of them in the world will become lessened. Almost all diseases, especially those of the nervous system, have predisposing and exciting causes. A man may go through life with a certain predisposition to gout, rheumatism, or insanity, and may be so fortunate or so wise as to escape them entirely as actualities, through avoidance of their exciting causes. If his children and grandchildren did the same, we are justified in supposing that the predisposition would grow weaker, and the exciting causes would need to be stronger to bring out any of those diseases. Nature always aims at a physiological ideal. Many diseases either tend to kill the individual and the tainted family, if the predisposition is strong enough, or they are slowly eradicated by this natural law of reversion to the ideal state. Between the one law and the other, insanity and many other nervous diseases should diminish in future generations, if we can lessen the exciting causes. Then the mere fact that most of the insane are now placed in hospitals diminishes a former cause of insanity by direct propagation when they wandered about the country and frequently bore children.

